# Investigation of Japanese encephalitis virus as a cause of acute encephalitis in southern Pakistan, April 2015–January 2018

**DOI:** 10.1371/journal.pone.0234584

**Published:** 2020-06-12

**Authors:** Tazeen Fatima, Abida Rais, Erum Khan, Susan L. Hills, Trudy V. Chambers, Aneeta Hotwani, Shahida Qureshi, Saad Shafquat, Saima Malik, Farah Qamar, Fatima Mir, Anthony A. Marfin, Anita Zaidi, Asif Raza Khowaja, Sadia Shakoor

**Affiliations:** 1 Departments of Pathology, Pediatrics, and Medicine, Aga Khan University, Karachi, Pakistan; 2 Arboviral Diseases Branch, Division of Vector-Borne Diseases, Centers for Disease Control and Prevention, Fort Collins, CO, United States of America; 3 Vaccine Introduction and Impact, Center for Vaccine Innovation and Access, PATH Seattle, Washington, United States of America; 4 Department of Obstetrics and Gynaecology, University of British Columbia, Vancouver, Canada; Faculty of Science, Ain Shams University (ASU), EGYPT

## Abstract

**Background:**

Japanese encephalitis (JE) occurs in fewer than 1% of JE virus (JEV) infections, often with catastrophic sequelae including death and neuropsychiatric disability. JEV transmission in Pakistan was documented in 1980s and 1990s, but recent evidence is lacking. Our objective was to investigate JEV as a cause of acute encephalitis in Pakistan.

**Methods:**

Persons aged ≥1 month with possible JE admitted to two acute care hospitals in Karachi, Pakistan from April 2015 to January 2018 were enrolled. Cerebrospinal fluid (CSF) or serum samples were tested for JEV immunoglobulin M (IgM) using the InBios JE *Detect*^TM^ assay. Positive or equivocal samples had confirmatory testing using plaque reduction neutralization tests.

**Results:**

Among 227 patients, testing was performed on CSF in 174 (77%) and on serum in 53 (23%) patients. Six of eight patient samples positive or equivocal for JEV IgM had sufficient volume for confirmatory testing. One patient had evidence of recent West Nile virus (WNV) neurologic infection based on CSF testing. One patient each had recent dengue virus (DENV) infection and WNV infection based on serum results. Recent flavivirus infections were identified in two persons, one each based on CSF and serum results. Specific flaviviruses could not be identified due to serologic cross-reactivity. For the sixth person, JEV neutralizing antibodies were confirmed in CSF but there was insufficient volume for further testing.

**Conclusions:**

Hospital-based JE surveillance in Karachi, Pakistan could not confirm or exclude local JEV transmission. Nonetheless, Pakistan remains at risk for JE due to presence of the mosquito vector, amplifying hosts, and rice irrigation. Laboratory surveillance for JE should continue among persons with acute encephalitis. However, in view of serological cross-reactivity, confirmatory testing of JE IgM positive samples at a reference laboratory is essential.

## Introduction

Flaviviral encephalitis is an emerging global problem [1, 2]. Notable among flaviviral encephalitides are infections due to Japanese encephalitis virus (JEV), West Nile virus (WNV), dengue virus (DENV), St Louis encephalitis virus (SLEV), and Murray Valley encephalitis virus. In South Asia, DENV is endemic in almost all countries, JEV transmission is widespread, and limited WNV transmission has been reported, most frequently from India [3, 4, 5].

Japanese encephalitis (JE) is a vaccine-preventable infection. Most human infections are asymptomatic; symptoms manifest as acute febrile illness (AFI), and in approximately 1/300 cases, with meningoencephalitis or acute flaccid paralysis [6]. Catastrophic sequelae including death and neuropsychiatric disability are observed [1]. According to the World Health Organization (WHO), borders of the region of endemicity for JE extend from Western Pacific islands in the east to Sindh province in southern Pakistan in the west [7]. Evidence of JE from Pakistan dates back to the early 1980s and 1990s, when studies were conducted in Karachi; serosurvey data and JEV ribonucleic acid detection in the cerebrospinal fluid (CSF) of an encephalitis patient suggested a low incidence of JE [8, 9]. More recent data on flaviviral and, more specifically, JE etiology of acute encephalitis from Pakistan are lacking. In 2018, Khan et al reported substantial cross-reactivity with DENV and WNV as the likely cause of JEV immunoglobulin M (IgM) detection in sera of patients with AFI in southern Pakistan [10].

It has been estimated that only about 10% of JE cases occurring annually in JE endemic areas are reported to WHO [5]. Therefore, there is a need to enhance surveillance systems, establish diagnostic infrastructure to confirm infections, and ensure routine case reporting occurs. During the last 15 years, the greater availability of SA 14-14-2 JE vaccine and recognition that it is an effective intervention to reduce JE deaths and the catastrophic sequelae among survivors has resulted in countries working to better understand their JE disease burden and to weigh the value of JE control efforts through the support of several organizations, including the Bill & Melinda Gates Foundation [11, 12, 13]. In Pakistan, there are very limited data available on JEV transmission, the risk for outbreaks of JE and other flaviviral diseases is unknown, and systematic acute encephalitis surveillance is lacking. The objective of this study was to investigate JEV as a cause of acute encephalitis in southern Pakistan.

## Materials and methods

### Study location

Laboratory diagnostic specimens were collected from patients with possible infectious acute encephalitis admitted to two acute care hospitals in Karachi (The Aga Khan University Hospital, and the Aga Khan Hospital, Garden). The catchment areas for these hospitals are wide, as patients from several distant rural and urban areas seek treatment at these hospitals in addition to the local population of Karachi.

### Patient enrollment

From April 2015 to January 2018, possible JE cases were defined as persons aged ≥1 month admitted to acute care wards in two hospitals with fever (>38 ⁰C) and at least one of the following symptoms: new onset of altered level of consciousness >24 hours, lethargy, irritability, change in personality, seizures, meningismus, or other focal neurological signs (e.g., cranial or sensory nerve deficits, abnormal movements, weakness of one or more limbs). Patients with the following findings were excluded: alternative bacterial, non-flavivirus viral, or parasitic cause for meningoencephalitis, acute uncorrected dehydration, suspected hepatic/ uremic encephalopathy or hepatorenal syndrome, prolonged (> 3 months) undiagnosed systemic illness, known cerebrovascular etiology/ stroke, and central nervous system tumors.

Initial laboratory testing to inform routine clinical care where indicated (biochemical and microbiological analysis of samples) was carried out at the Aga Khan University (AKU) clinical laboratory. Testing for malaria was done by antigen detection using an immunochromatographic rapid test or by microscopic examination of a peripheral blood smear. CSF analyses for glucose, protein, and cell count and testing for herpes simplex virus (HSV) and *Mycobacterium tuberculosis* (MTB) using standard culture methods and/or molecular detection were conducted when CSF was available.

### Ethics approval and consent to participate

The study protocol was approved by the Ethics Review Committee of the Aga Khan University (3098-PED-ERC-14). All participants or their next of kin provided written consent for collection of cerebrospinal fluid or serum samples and testing for Japanese encephalitis virus antibody on samples for this study.

### Sample collection, management and diagnostic testing

If a lumbar puncture was clinically indicated and performed as part of routine care, CSF was archived after patient consent for JEV IgM testing. If a lumbar puncture was not performed, or if insufficient CSF sample remained after routine laboratory testing, a single serum sample was collected after written consent and archived for JEV IgM testing. Therefore, either CSF or serum samples (not both) were collected.

All serum and CSF samples were stored at -80⁰C at the AKU Infectious Disease Research Laboratory (IDRL), a biosafety level 2 facility, and batch-tested. Initial testing for JE IgM was carried out using a JE IgM antibody capture enzyme-linked immunosorbent assay (MAC-ELISA) with the InBios JE *Detect*^TM^ kit (InBios International Inc, Seattle, Washington, USA). CSF and serum samples were tested as per manufacturer instructions (i.e., sera tested with 1 in 100 dilution and CSF tested undiluted). Positive-to-negative absorbance ratios (P/N ratios) were recorded. As per manufacturer instructions, samples were classified as positive (P/N >10), equivocal (P/N 6–10), or negative (P/N <6). All samples with initial equivocal results were retested.

Samples testing positive or equivocal for JEV IgM were tested for DENV and WNV IgM using the InBios DENV *Detect*^TM^ IgM capture ELISA and the West Nile *Detect*^TM^ IgM capture ELISA kits as per manufacturer instructions. Samples testing positive or equivocal for JEV IgM with sufficient remaining sample for additional testing were shipped to the Centers for Disease Control and Prevention (CDC), Fort Collins, USA for confirmatory testing.

At CDC, CSF samples were tested for neutralizing antibody (NAb) against JEV and WNV using a 90% plaque reduction neutralization test (PRNT90). PRNT90 was performed by incubating serial dilutions of serum- or CSF-virus mixtures until the resulting viral culture showed a reduction in plaque count of 90% [14]; this dilution was reported as the neutralizing end-point titer. CSF samples with a reciprocal titer of 2 or greater in the WNV PRNT90 were tested with the CDC WNV/SLEV microsphere-based immunoassay (MIA) [14]. Serum samples were tested using the CDC WNV/SLEV MIA, dengue MAC-ELISA, and by PRNT90 to detect NAb against JEV, WNV, and DENV 1, 2, 3, and 4 [15, 16].

A confirmed acute infection with JEV, WNV, or DENV was defined as a positive IgM result for the respective virus and a PRNT90 reciprocal titer against that same virus that was at least fourfold higher than the reciprocal titer against the other tested viruses [17]. A sample with a positive IgM result for JEV, WNV or DENV but less than fourfold difference in reciprocal PRNT titers was considered an unspecified flavivirus infection.

### Data analysis

Data were entered and analyzed in MS Excel. Frequency, median and interquartile range (IQR) values were calculated, where applicable.

## Results

### Patient enrollment and clinical features

Of 227 patients enrolled in the study, 100 (44%) were children less than 18-years-old. The median age of the cohort was 24 years (IQR 53–7; 46). The male to female ratio was 0.6.

Median time from symptom onset to sample collection was 4 days (IQR 7–3; 4). Of 227 enrolled patients, clinical outcomes were available for 219 (96%). Of these 219 persons, 13 (6%) died due to the acute illness. Patient demographics, neurological manifestations, and results of JEV IgM testing for all enrolled cases are provided in S1 File (supporting information).

### Laboratory testing

A total of 174 CSF and 53 acute phase serum samples were available from 227 enrolled patients. Malaria was excluded for all 227 patients. Among the 174 patients for whom CSF samples were collected, HSV and MTB were excluded for all. Cross-tabulation of syndromic presentation, samples collected and laboratory tests performed as part of routine care is presented in S1 Table (supporting information).

On initial testing, eight (five CSF and three serum) samples were positive (n = 6) or equivocal (n = 2) for JEV IgM. Clinical, neurophysiological, radiological, and laboratory parameter details for these patients are shown in Table 1. JEV, DENV, and WNV IgM testing showed substantial cross-reactivity. Of the eight samples that were positive or equivocal for JEV, seven were positive for WNV IgM, two were positive for DENV IgM, and five were equivocal for DENV IgM (Table 1).

**Table 1:** Characteristics, clinicoradiological features, and flaviviral IgM and PRNT results in CSF and serum samples of eight patients positive for JE IgM on initial testing.

No.	Age (years), Sex (M/F)	Clinicoradiological and/ or neurophysiological features	Sample type tested	InBios MAC-ELISA			CDC ELISA/ MIA		PRNT_90_ titer			Final interpretation	Outcome
				JEV	DENV	WNV	DENV	WNV/SLEV	JEV	DENV1-4	WNV		
**1**	70, M	Meningoencephalitis; CT scan: age-related global involutional changes	CSF	+	E	+	NP	NP	NP	NP	NP	Unresolved	Recovered, discharged home
**2**	53, F	Encephalitis; MRI: chronic ischemic changes in periventricular white matter and basal ganglia	CSF	+	+	+	NP	+ (WNV)	2	NP	64	West Nile virus infection	Recovered, discharged home
**3**	60, F	Encephalitis; MRI: Nonspecific T2 hyperintense signals in bilateral basal ganglia and thalami	CSF	+	E	+	NP	NP	64	NP	128	Unspecified flaviviral infection	Recovered, discharged home
**4**	27, F	Meningoencephalitis and multiorgan dysfunction; CT scan: normal	CSF	E	E	+	NP	NP	16	NP	NP	Unresolved	Died
**5**	63, F	Encephalopathy; MRI: periventricular deep white matter ischemic changes; EEG: slow background rhythm	CSF	+	-	-	NP	NP	NP	NP	NP	Unresolved	Recovered, discharged home
**6**	30, M	Fever with irritability; no imaging studies	Serum	E	E	+	+	-	320	DENV 2 >40960	80	Dengue virus infection	Recovered, discharged home
**7**	24, M	Fever, headache, and irritability; CT scan: normal	Serum	+	E	+	+	+ (WNV)	20	DENV 1–4 <10	320	West Nile virus infection	Recovered, discharged home
**8**	15, M	Encephalitis; CT scan: normal; EEG: quasi-periodic generalized high voltage spike sharp and slow waves	Serum	+	+	+	+	E (WNV)	1280	DENV 3 2560	640	Unspecified flaviviral infection	Recovered, discharged home

M = male; F = female; CSF = cerebrospinal fluid; PRNT_90_ = Plaque reduction neutralization test with 90% endpoint; CT = computerized tomography; MRI = Magnetic Resonance Imaging; MAC-ELISA = IgM antibody capture enzyme linked immunosorbent assay; JEV = Japanese encephalitis virus; DENV 1–4 = Dengue virus serotypes 1–4; WNV = West Nile virus; SLEV = St. Louis encephalitis virus; (+) = Positive; (-) = Negative; NP = not performed; E = Equivocal; EEG = Electroencephalogram.

Of the eight samples with positive or equivocal JEV IgM testing, six were tested at CDC. CSF testing for Patient 2 with clinical encephalitis (Table 1) confirmed a recent neurologic WNV infection. Serum testing for two patients (6 and 7 in Table 1) were confirmed as recent DENV and WNV infections; neither had CSF available for testing so neurologic infection could not be confirmed. Two patients (Patients 3 and 8) were classified as unspecified flavivirus infections based on CSF and serum results, respectively. Patient 4 had equivocal JEV IgM and positive WNV IgM results, and JEV NAb in the CSF, but insufficient sample to test for WNV NAb; classification was not possible.

## Discussion

In Pakistan, JE diagnosis is complicated due to the possibility of infection from other endemic co-circulating flaviviruses. Our study in hospitalized patients with possible infectious acute encephalitis from April 2015 to January 2018 could not confirm or exclude local JEV transmission. While one DENV and two WNV infections were confirmed, a specific etiology for the other five patients with JEV IgM antibody could not be determined because of insufficient sample for further testing or because testing only revealed an unspecified flavivirus infection. Cross-reactive antibody to DENV and WNV, two flaviviruses known to cause endemic disease in Pakistan [10,18], was the biggest challenge to diagnosis.

Several factors, including changes in agricultural practices and climate, migratory bird patterns, and population shifts, may increase the risk of endemic JE in the future [19, 20, 21]. Although acute encephalitis surveillance in two urban hospitals in Karachi was unable to confirm any recent JE cases, the presence of *Culex tritaeniorhynchus* and related species [22, 23] and the intense rice production in the region increase this likelihood [23, 24, 25]. Previous and current research on JE in Pakistan has been conducted in Karachi and surrounding areas in southern Pakistan, and investigation of possible transmission in other, particularly more rural, areas would be useful. Furthermore, more current entomological surveillance data for Pakistan are needed, and efforts should be made to isolate JEV from known vector mosquitoes.

Arboviral etiologies should be considered in the differential diagnosis for all persons with acute encephalitis when the geographic or exposure history suggests possible arboviral illness [26]. JE, dengue, and WNV disease can be clinically indistinguishable in persons presenting with AFI or acute encephalitis. Given the high incidence of dengue, ongoing evidence of WNV transmission, and limited but past evidence of JEV transmission in Pakistan, patients with acute encephalitis of unknown etiology should be tested for WNV, JEV, and DENV IgM in both CSF and serum samples, with convalescent serum testing at 10 days. For WNV and JEV, low viral titers in blood and CSF and typically high neutralizing antibodies at the time of presentation mean molecular or virus detection methods are usually unhelpful, so IgM detection followed by PRNT to confirm the infecting virus remains the gold standard for confirmatory diagnosis [27, 28]. We therefore employed serologic tests to determine the possible presence of JEV infection in our study, despite the known challenges of cross-reactivity in serologic testing. Molecular diagnostic approaches would provide greater specificity, and recent developments in tests with greater sensitivity have shown promise, but these tests are still insufficiently sensitive for routine diagnostic purposes [27, 28, 29]. Systematic and reliable laboratory testing will be essential to identify transmission and emergence of JEV in Pakistan. Ideally, reference laboratory capacity for nucleic acid amplification testing and next-generation sequencing should be developed for flaviviruses and other viral neuropathogens such as Nipah virus, the non-polio enteroviruses (e.g., EV-71), and other viruses that cause encephalitis outbreaks.

Prevention and control of flaviviral infections centers on mosquito vector control, prevention of exposure among humans, and vaccination when available [30]. Several JE vaccines are currently licensed and used in many parts of the world, and the WHO recommends JE vaccination be integrated into national immunization programs in all areas where JE is recognized as a public health priority [13, 31]. Based on our results, a JE vaccination program is not currently warranted for southern Pakistan, and vaccine is not recommended for most travelers visiting southern Pakistan. However, given the very limited data available, JE vaccine may be considered for high risk-travelers such as entomologists, wildlife enthusiasts, or other travelers spending prolonged periods of time visiting agricultural lands and farms. Irrespective of vaccination, all travelers to Pakistan should be advised to take precautions to avoid mosquito bites to reduce the risk for vector-borne diseases.

Our study has several limitations. It was not a population-based study, and therefore cannot provide incidence of acute encephalitis or encephalitis due to flaviviruses. The hospital-based surveillance also captured a lower proportion of children than adults, which is potentially a concern given that JE is mainly seen in children in JE-endemic countries. Surveillance was not conducted nationwide, so flavivirus transmission in geographical areas outside the catchment area of the hospitals was not assessed. Other approaches to identify endemic JE transmission such as serosurveys may be less useful because of substantial antibody cross-reactivity to DENV and WNV, two locally endemic flaviviruses. We were unable to obtain convalescent serum samples from patients which would have assisted with diagnosis. DENV PRNT testing was not performed on CSF samples although DENV can occasionally cause neurologic infection. Finally, insufficient sample volumes prevented complete testing for several samples.

## Conclusions

Sentinel surveillance for JE from April 2015 to January 2018 performed in two hospitals in Karachi in southern Pakistan was unable to confirm JEV as a cause of acute encephalitis. Surveillance for acute encephalitis due to flaviviruses in Pakistan should be continued because of known endemic transmission of DENV and WNV, past evidence of limited JEV transmission, the presence of the appropriate vectors and amplifying hosts, and with reports of JEV outbreaks in India to the east of Pakistan [32]. This is especially important because multiple JE vaccines and newly licensed dengue vaccines are available. Additionally, surveillance systems need strengthening in Pakistan through liaison between clinical and public health systems, and peripheral and reference laboratories, to identify important emerging flaviviral illnesses in the region.

## Supporting information

S1 File Demographics, manifestations, and JEV IgM. This file contains patient demographics, neurological manifestations, and results of JEV IgM testing for all enrolled cases.
(XLSX)

S1 Table Laboratory tests for clinical care in study patients. This table presents cross-tabulation of syndromic presentation, samples collected, and laboratory tests performed as part of routine care among study patients.
(DOCX)
