# Supplementary material for: Investigation of Japanese encephalitis virus as a cause of acute encephalitis in southern Pakistan, April 2015–January 2018
Source: PLoS One. 2020 Jun 12;15(6):e0234584. doi: 10.1371/journal.pone.0234584 (PMC7292402; doi:10.1371/journal.pone.0234584)
Supplement: S1 Table — This table presents cross-tabulation of syndromic presentation, samples collected, and laboratory tests performed as part of routine care among study patients. (DOCX) [file pone.0234584.s002.docx]

| Study Sample & test panels performed | Clinical presentation of acute encephalitis | | Total |
| --- | --- | --- | --- |
| CSF | AFI with Altered mental status | AFI with acute flaccid paralysis or lethargy/ headache/ irritability |  |
| A | - |  |  |
| B | 13 | 1 | 14 |
| C | 45 | 2 | 47 |
| D | 63 | 6 | 69 |
| E | 9 | 1 | 10 |
| F | 28 | 6 | 34 |
| Subtotal | 158 | 16 | 174 |
|  |  |  |  |
| Serum |  |  |  |
| A | 12 | 3 | 15 |
| B | 3 | 1 | 4 |
| C | 4 |  | 4 |
| D | 14 | 2 | 16 |
| E | 3 | 3 | 6 |
| F | 7 | 1 | 8 |
| Subtotal | 43 | 10 | 53 |
|  |  |  |  |
| TOTAL | 201 | 26 | 227 |

Panel A= Blood test for malaria (Immuochromatographic ICT or peripheral smear *2) + Blood cultures

Panel B= Panel A tests + CSF analysis and culture

Panel C= Panel A & B tests + HSV I and II PCR

Panel D= Panel A & B tests + Tuberculosis PCR/ Xpert MTB/RIF®

Panel E= Panel A & B tests + Filmarray® ME panel test

Panel F= Panel A & B tests + Filmarray® ME panel test + Tuberculosis PCR/ Xpert MTB/RIF

TB PCR= PCR (Xpert MTB/RIF or other) for Mycobacterium tuberculosis; HSV PCR= PCR for Herpes Simplex Virus I and II; Filmarray® commercial test for 14 meningoecephalitis pathogens (Escherichia coli K1, Haemophilus influenzae, Listeria monocytogenes, Neisseria meningitidis, Streptococcus agalactiae, cytomegalovirus (CMV), enterovirus (EV), herpes simplex virus 1 (HSV-1), HSV-2, human herpesvirus 6 (HHV-6), human parechovirus (HPeV), varicella-zoster virus (VZV), and Cryptococcus neoformans/Cryptococcus gattii)

AFI= Acute febrile illness; CSF= Cerebrospinal fluid; PCR= Polymersase Chain Reaction
